# What makes a blood cell based miRNA expression pattern disease specific? - A miRNome analysis of blood cell subsets in lung cancer patients and healthy controls

**DOI:** 10.18632/oncotarget.2419

**Published:** 2014-09-19

**Authors:** Petra Leidinger, Christina Backes, Indra N. Dahmke, Valentina Galata, Hanno Huwer, Ingo Stehle, Robert Bals, Andreas Keller, Eckart Meese

**Affiliations:** ^1^ Institute of Human Genetics, Medical School, Saarland University, Building 60, 66421 Homburg/Saar, Germany; ^2^ Department of Clinical Bioinformatics, Saarland University, Building E2.1, 66123 Saarbrücken, Germany; ^3^ Institute for Clinical and Experimental Surgery, Saarland University, Building 65, 66421 Homburg/Saar, Germany; ^4^ Department of Thoracic Surgery, Voelklingen Heart Center, 66333 Voelklingen Germany; ^5^ Department of Pneumology, Medical School, Saarland University, Building 91, 66421 Homburg/Saar, Germany

**Keywords:** microRNA, leukocytes, lung cancer, microarray, cell separation

## Abstract

There is evidence of blood-borne miRNA signatures for various human diseases. To dissect the origin of disease-specific miRNA expression in human blood, we separately analyzed the miRNome of different immune cell subtypes, each in lung cancer patients and healthy individuals. Each immune cell type revealed a specific miRNA expression pattern also dependinging on the cell origin, line of defense, and function. The overall expression pattern of each leukocyte subtype showed great similarities between patients and controls. However, for each cell subtype we identified miRNAs that were deregulated in lung cancer patients including hsa-miR-21, a well-known oncomiR associated with poor lung cancer prognosis that was up-regulated in all leukocyte subtype comparisons of cancer versus controls. While the miRNome of cells of the adaptive immune system allowed only a weak separation between patients and controls, cells of the innate immune system allowed perfect or nearly perfect classification. Leukocytes of lung cancer patients show a cancer-specific miRNA expression profile. Our data also show that cancer specific miRNA expression pattern of whole blood samples are not determined by a single cell type. The data indicate that additional blood components, like erythrocytes, platelets, or exosomes might contribute to the disease specificity of a miRNA signature.

## INTRODUCTION

MicroRNAs (miRNAs) are tiny molecules (around 20 nucleotides) with huge impact on the function of the single cell, but also the complete organism, as they play an important role in regulation of gene expression in physiological and pathophysiological conditions. So far, the miRNA Database (miRBase Release 20, http://www.mirbase.org/, [1, 2]) contains more than 24,500 miRNA entries, including 2,578 human mature miRNAs. The influence of the miRNome becomes clear with regard to the huge number of genes that are regulated by these miRNAs [3]. Changes in miRNA expression can be driven by the onset of a disease or vice versa the disease onset is driven by altered miRNA expression due to external stimuli [4–7]. The first is a good starting point for the identification of disease-related biomarkers. There are a vast number of studies identifying disease specific miRNAs in tissue but a comparatively lower number of studies based on blood. In our previous studies we investigated the miRNA expression pattern in blood samples from patients suffering from different non-cancer and cancer diseases, including lung cancer [8–14]. Lung cancer accounts for more deaths than any other cancer in both men and women. In USA the estimated number of new lung cancer cases for 2013 is 228,190 and the number of deaths is 159,480 for men and women combined [15]. As the minority of lung cancer cases is diagnosed at early stages, when the disease is still localized, the screening of an asymptomatic population at high risk for lung cancer is able to reduce the cancer related mortality. Blood based analysis methods offer themselves as non-invasive tools for screening of high-risk individuals. In our former studies, we showed that whole blood samples of lung cancer patients can be separated from those of healthy control individuals with high accuracies between 87% and 95% depending on the set of differentially expressed miRNAs used for classification [10, 13]. However, as blood is a complex mixture of specialized cell types with a multitude of functions it would be interesting which type of blood cells is significantly involved in cancer-specific miRNA expression patterns of whole blood samples. In the recent past, cell type specific miRNA expression patterns in different immune cell subsets were detected for healthy subjects. Allantaz et al. analyzed the miRNA content of nine immune cell subsets and identified cell type specific miRNAs. Moreover, they identified a regulatory relationship between the miRNA and the mRNA expression pattern [16]. Merkerova et al. investigated the expression of a couple of miRNAs in reticulocytes, platelets, granulocytes, monocytes, B-cells, and T-cells, and showed that a small number of miRNAs is sufficient for a perfect clustering of the different blood cell subsets [17]. In a mouse model, Petriv et al. even isolated 27 phenotypically distinct cell populations from mouse hematopoietic tissues and found similar miRNA expression pattern due to cell lineage relations and functional similarities [18]. However, to the best of our knowledge, there is no study investigating the miRNA expression pattern of specific immune cell subsets in patients with cancers other than leukemia.

Here, we analyzed the miRNA expression patterns of five different blood cell subsets, including eosinophilic and neutrophilic granulocytes (CD15+), monocytes (CD14+), B-cells (CD19+), T-cells (CD3+), and natural killer (NK) cells (CD56+) and compared the respective expression patterns between lung cancer patients and healthy controls. We have chosen these cell populations as NK cells and neutrophilic granulozytes are the first cells of the innate immune system invading the cancer surrounding, followed by dentritic cells as well as cells of the adaptive immune system, including B-cells and T-cells. Close interaction of those cells of the innate and adaptive immune system is required for effective immune reaction as a primary defense against cancer (also reviewed by Matejuk et al. [19]). The main goal of our study is to contribute to a deeper understanding of miRNA expression changes in certain blood cells driven by the onset of lung cancer.

## RESULTS

### Purity of cell sorting

We isolated different leukocyte subpopulations either with antibody-coupled non-magnetic or magnetic beads depending on the cell type. The purity of the cell sorting was examined by flow cytometric measurements. With our newly established protocol to minimize the amount of blood necessary to isolate enough cells per subtype for microarray analyses we reached a median purity for the different cell types of 84.90%. In detail, the purity was 84.90% for CD3+, 60.82% for CD19+, 87.19% for CD15+, 77.62% for CD14+, and 85.97% for CD56+ cells.

### Overall detected miRNAs in whole blood and blood leukocytes

Out of 1,205 investigated miRNAs a total of 671 were not detected in any of the analyzed leukocyte subsets or whole blood samples from healthy controls and lung cancer patients. In whole blood samples 319 and 394 miRNAs were expressed in at least one control or lung cancer sample, respectively. Between 268 and 400 miRNAs were detected in at least one sample per cell subset of either lung cancer samples or controls with the least miRNAs in CD19+ B-cells and the most in CD15+ granulocytes. Between 113 and 284 miRNAs were detected in all analyzed samples per cell subset of either lung cancer samples or controls with again the least miRNAs in CD19+ B-cells and the most in CD15+ granulocytes.

**Figure 1:**
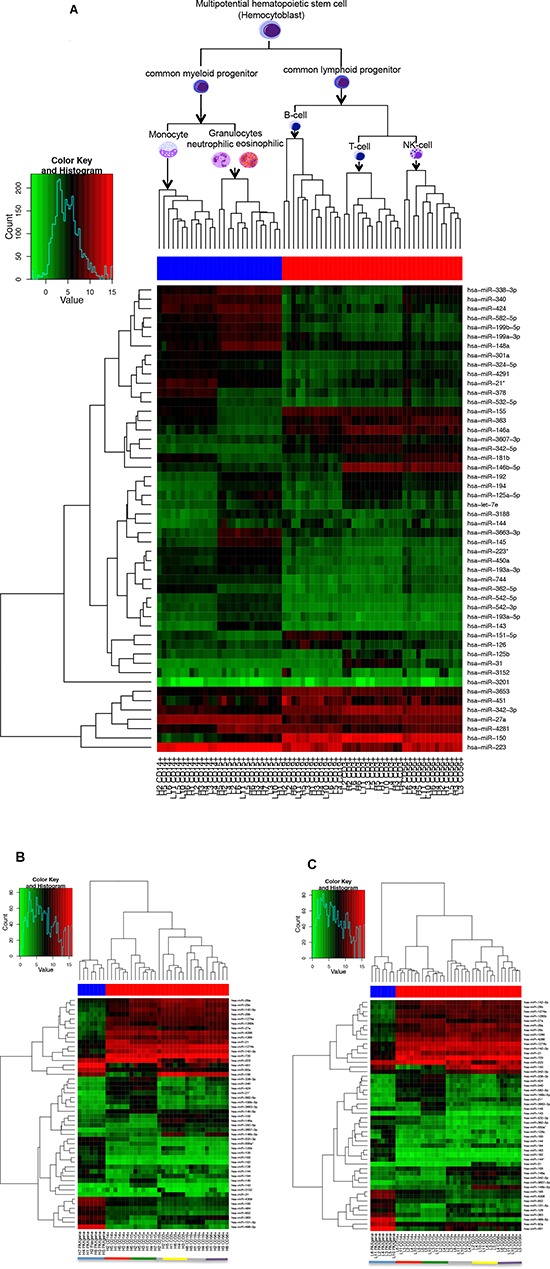
Heatmap using the 50 miRNAs with the highest variance over all analyzed samples Figure **(A)** shows the heatmap for all samples analyzed together. The color of the bar under the dendrogram indicates the cells with common myeloid (blue) and common lymphoid (red) progenitor. Figure **(B)** shows the heatmap for the healthy individuals and Figure **(C)** for the lung cancer patients. Here the color of the bar under the dendrogram indicates whole blood samples (blue) and the single immune cell subtypes (red). The single cell types are also indicated by the coloured bar at the bottom of the figure (blue=whole blood, red=monocytes CD14+, green=granulocytes CD15+, grey= B-cells CD19+, yellow=T-cells CD3+, purple=NK-cells CD56+).

However, in general, there seems to be a stable miRNA pattern that differentiates between the different blood cell types. When using the 50 miRNAs with the highest variance over all samples, we can cluster the cell fractions perfectly according to their original cell type (see Figure 1A).

### Comparison of whole blood and leukocyte subsets

Regarding only the detected miRNAs, independent of the expression values, the majority of miRNAs that was found in all leukocyte subpopulations was, with minor exceptions, also found in whole blood samples. In whole blood samples of healthy controls we found nine miRNAs (hsa-miR-130b*, hsa-miR-182, hsa-miR-183, hsa-miR-3180-3p, hsa-miR-3200-5p, hsa-miR-409-3p, hsa-miR-4318, hsa-miR-501-5p, hsa-miR-942) that were detected in all whole blood samples but not in any of the separated leukocyte subsets of healthy controls. For the lung cancer patients five miRNAs (hsa-miR-3180-3p, hsa-miR-3200-5p, hsa-miR-4318, hsa-miR-942, hsa-miR-144*) were detected in all whole blood samples but not in any of the separated leukocyte subsets of lung cancer patients. On the other hand, we identified three miRNAs for healthy controls (hsa-miR-3607-3p, hsa-miR-181d, hsa-miR-21*) and three miRNAs for lung cancer patients (hsa-miR-3607-3p, hsa-miR-21*, hsa-miR-34a) that were detected in >90% of the leukocyte samples but not in any whole blood sample, respectively. However, looking at the expression values whole blood showed a completely different expression pattern than the single cell fractions (Figure 1 B and C). Here, there was a clear clustering into the leukocytes and whole blood.

In order to investigate, whether the expression pattern of the whole blood sample can be predicted from the expression levels of the miRNAs in the leukocyte cell subsets by a linear model, we computed the estimates for each patient separately using linear regression implemented in the R build-in package “stats”. The results demonstrated acceptable estimates only for miRNAs with relatively low expression levels, while the remaining predicted values showed a noticeable bias. However, the Pearson's correlation coefficient computed for each patient and all miRNAs for each cell subset compared to whole blood, revealed a moderate linear dependence (0.79 <= rho <= 0.84) between these features. This suggests, that either the dependence between the whole blood samples and the subsets cannot be captured by means of a linear model, or there are some other factors (e.g. other blood components) contributing to the expression pattern of the miRNAs.

### Leukocyte subset specific miRNA expression

**Figure 2:**
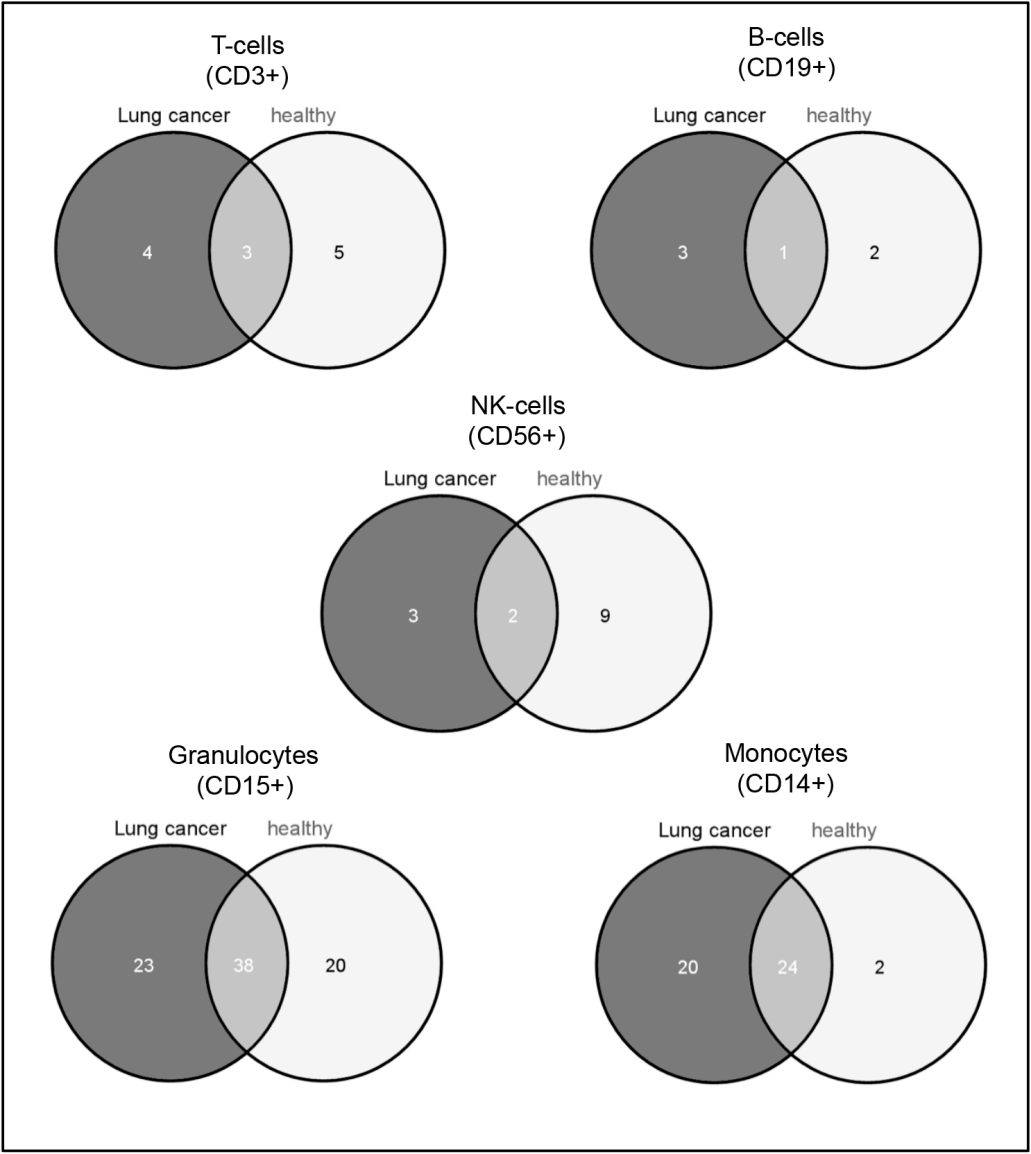
Venn diagram showing the leukocyte subset specific miRNAs The dark grey circle shows the cell subset specific miRNAs in lung cancer samples and the light grey circle shows the cell subset specific miRNAs in control samples. The overlap indicates the miRNAs that were found to be subtype specific in both sample groups. A list of the miRNAs is given in Supplemental Table 1.

To identify blood cell type specific miRNAs, we investigated the miRNA expression pattern of all samples from lung cancer patients and all samples from healthy individuals separately. To this end, we computed all pairwise comparisons between the leukocyte subtypes for samples from healthy individuals and lung cancer patients separately and built the intersections of deregulated miRNAs according to the adjusted t test (p-value < 0.05) to collect the cell subset specific miRNAs. To exclude overlaps between the specific miRNAs of each subtype, we removed those miRNAs from each leukocyte subtype specific set that are contained in any other cell specific set. After that, we found in the CD14+ cell population 44 specific miRNAs for lung cancer patients and 26 specific miRNAs for controls. In the CD3+ cell subset we found 7 lung cancer specific miRNAs and 8 miRNAs specific for controls. Only 4 miRNAs were specific for lung cancer and 3 specific for controls in the CD19+ cells, and for the CD56+ cells 5 miRNAs were specific for lung cancer and 11 for controls. For CD15+ cells we found the most specific miRNAs with 61 miRNAs for lung cancer patients and 58 for healthy controls. An overview of all specific miRNAs for lung cancer and controls is shown in Supplemental Table 1. Venn diagrams showing the overlap between the specific miRNAs in the different groups are shown in Figure 2.

### Comparison of the entire miRNome of single leukocyte subsets between lung cancer patients and healthy individuals

We compared the miRNA expression pattern of the five different immune cell populations isolated either from blood of lung cancer patients or from blood of healthy individuals. After quantil normalization and Benjamini-Hochberg adjustment for multiple testing, we found no significant p-values <0.05 for any comparison [20, 21]. However, we filtered out all miRNAs with raw p-values <0.05 that were expressed in at least all samples of one group, i.e., either in all samples from lung cancer patients or in all samples from healthy control individuals, to get a first idea if the analyzed immune cell subpopulations contribute to the lung cancer specific miRNA expression pattern. In CD3+ cells we found 13 miRNAs, in CD19+ cells 5 miRNAs, in CD15+ cells 12 miRNAs, in CD14+ cells 15 miRNAs, and in CD56+ cells 11 miRNAs with raw p-value <0.05. The comparison of total blood of the same patients and controls (PAXgene RNA blood tubes) revealed 24 deregulated miRNAs (see Table 1). Interestingly, we found only slight overlap in the deregulated miRNAs between the whole blood samples and the single immune cell subsets. Only hsa-miR-21 was up-regulated in all cell subsets and in whole blood of lung cancer patients compared to the healthy controls. Four other miRNAs (hsa-miR-342-5p, hsa-miR-194, hsa-miR-150, hsa-miR-132) were deregulated in the same and two miRNAs (hsa-miR-22*, hsa-miR-30e*) were deregulated in the opposite direction in whole blood and in one other cell subset.

**Table 1:** Union of deregulated miRNAs (raw p-value <0.05) between control and lung cancer samples For each comparison and the deregulated miRNAs of this comparison the raw p-values are listed in this Table. Red font means down-regulation in lung cancer and green font means up-regulation in lung cancer. The last column gives the number of comparisons were the respective miRNA is deregulated. The last line gives the total number of deregulated miRNAs per comparison.

	control whole blood vs lung cancer whole blood	control cd3+ vs lung cancer cd3+	control cd56+ vs lung cancer cd56+	control cd19+ vs lung cancer cd19+	control cd15+ vs lung cancer cd15+	control cd14+ vs lung cancer cd14+	overlaps
hsa-miR-21	0.01418	0.01421	0.01244	0.04076	0.02069	0.01396	6
hsa-miR-21*	-	0.00313	0.03142	-	0.02400	0.00007	4
hsa-miR-451	-	-	-	-	0.01037	0.02953	2
hsa-miR-132	0.04971	-	0.04312	-	-	-	2
hsa-miR-22*	0.01423	-	-	-	-	0.02099	2
hsa-miR-30b	-	0.03085	-	-	-	0.00476	2
hsa-miR-194	0.03964	-	-	-	0.03493	-	2
hsa-miR-342-5p	0.00543	-	-	-	0.01298	-	2
hsa-miR-3653	-	-	0.02964	-	-	0.01418	2
hsa-miR-29a	-	-	-	0.03588	-	0.02344	2
hsa-miR-939	-	0.03551	-	-	0.03670	-	2
hsa-miR-150	0.04912	-	-	0.02248	-	-	2
hsa-let-7d	-	-	-	-	0.02803	0.01666	2
hsa-miR-30e*	0.03244	-	0.03755	-	-	-	2
hsa-miR-342-3p	-	-	-	-	-	0.00137	1
hsa-miR-365	-	-	-	-	-	0.00123	1
hsa-miR-4270	0.00388	-	-	-	-	-	1
hsa-miR-125b	-	0.00925	-	-	-	-	1
hsa-miR-223	0.03646	-	-	-	-	-	1
hsa-miR-139-3p	0.02598	-	-	-	-	-	1
hsa-miR-769-3p	-	-	-	-	0.02682	-	1
hsa-miR-27a	0.04996	-	-	-	-	-	1
hsa-miR-24	0.00041	-	-	-	-	-	1
hsa-miR-181a-2*	-	-	0.01788	-	-	-	1
hsa-miR-30a	0.03166	-	-	-	-	-	1
hsa-miR-30c	-	-	-	-	-	0.02503	1
hsa-miR-30d	-	-	-	-	-	0.01230	1
hsa-miR-1274a	-	-	-	0.04519	-	-	1
hsa-miR-3607-3p	-	-	0.02960	-	-	-	1
hsa-miR-19b	-	0.02934	-	-	-	-	1
hsa-miR-362-5p	-	-	-	-	-	0.01651	1
hsa-miR-4291	-	-	-	-	0.03070	-	1
hsa-miR-1246	-	-	-	0.03008	-	-	1
hsa-miR-454*	0.02529	-	-	-	-	-	1
hsa-miR-126	-	-	-	-	0.03525	-	1
hsa-miR-34a	-	0.00469	-	-	-	-	1
hsa-miR-301a	-	-	0.04892	-	-	-	1
hsa-miR-3665	0.01287	-	-	-	-	-	1
hsa-miR-99a	-	0.01726	-	-	-	-	1
hsa-miR-1275	0.03231	-	-	-	-	-	1
hsa-miR-130a	-	-	-	-	0.00514	-	1
hsa-miR-424	-	0.02316	-	-	-	-	1
hsa-miR-942	0.00262	-	-	-	-	-	1
hsa-miR-93*	0.01108	-	-	-	-	-	1
hsa-miR-31	-	0.01306	-	-	-	-	1
hsa-miR-16-2*	0.02083	-	-	-	-	-	1
hsa-miR-572	0.03918	-	-	-	-	-	1
hsa-miR-18a	-	-	-	-	-	0.01648	1
hsa-miR-361-3p	0.01000	-	-	-	-	-	1
hsa-miR-181a	-	0.04819	-	-	-	-	1
hsa-miR-181c	-	-	0.01246	-	-	-	1
hsa-miR-155	-	-	-	-	-	0.02947	1
hsa-miR-550a*	0.04339	-	-	-	-	-	1
hsa-miR-326	0.02127	-	-	-	-	-	1
hsa-miR-15b	-	0.01265	-	-	-	-	1
hsa-miR-28-5p	-	-	0.04491	-	-	-	1
hsa-let-7g	-	-	0.04580	-	-	-	1
hsa-let-7i	0.00369	-	-	-	-	-	1
hsa-miR-744	-	-	-	-	0.01262	-	1
hsa-miR-26a	-	0.02901	-	-	-	-	1
**number of deregulated miRNAs**	**24**	**13**	**11**	**5**	**12**	**15**	

### Leukocyte subpopulations of controls and lung cancer patients can be classified by their miRNome

We performed classification using Support Vector Machine with linear kernel, leave-one-out cross-validation, 20 repetitions and the above mentioned miRNAs with raw p-values <0.05 that were expressed in at least all samples of one group per comparison (see also Table 1). Classification reached accuracies from 69.23% (for CD3+ cells) to 100% (for CD15+ cells). The detailed classification results are listed in Table 2.

**Table 2:** Classification results Classification results for control immune cell subtypes vs lung cancer immune cell subtypes with linear kernel, leave-one-out cross-validation, and the deregulated miRNAs per comparison with raw p-values <0.05 (listed in Table 2).

	accuracy, mean	specificity, mean	sensitivity, mean
CD3+ normal vs lung cancer	0.6923	0.7143	0.6667
CD19+ normal vs lung cancer	0.7692	0.8571	0.6667
CD15+ normal vs lung cancer	1.0	1.0	1.0
CD14+ normal vs lung cancer	0.9231	0.8571	1.0
CD56+ normal vs lung cancer	0.9231	0.8571	1.0
whole blood normal vs lung cancer	0.9286	1.0	0.8571

### miRNA expression pattern is indicative for the blood cell origin, line of defense, and function

The heatmap of Figure 1 revealed not only a cell type specific miRNA expression pattern, but according to the dendrogram it is also evident that there is a specific miRNA expression pattern indicative for the cellular origin and function and the line of defense. To further analyze this, we grouped the cell populations of cancer patients and controls either according to their origin (myeloid or lymphoid progenitors), according to their line of defense (innate or adaptive immune system), and according to their function (antigen presenting cells or cytotoxic cells). We filtered out only those miRNAs that were expressed in at least all samples of one group of the respective comparison and that showed a significant p-value <0.05 after Benjamini-Hochberg adjustment. We first compared the significant miRNAs identified in the comparison of cells derived from myeloid progenitors (CD15+, CD14+) versus cells derived from lymphoid progenitors (CD3+, CD19+, CD56+) of lung cancer patients and controls. For the cancer patients we found 75 significant miRNAs and for the controls 64 significant miRNAs. The overlap was between 60 miRNAs that were also deregulated in the same direction in both groups. Interestingly, 4 miRNAs were only deregulatedn in the control group and 15 miRNAs were exclusively deregulated in the lung cancer group between cells derived from myeloid and lymphoid progenitors.

The same analysis as mentioned above was done for the cell populations from the innate (CD15+, CD14+, CD56+) versus adaptive (CD3+, CD19+) immune system. Here, we found 130 miRNAs significantly deregulated in lung cancer blood samples and 115 miRNAs in blood samples from healthy individuals, with an overlap of 106 identically deregulated miRNAs. A total of 24 miRNAs was exclusively found in lung cancer samples, and 9 miRNAs were only identified in control samples.

**Figure 3:**
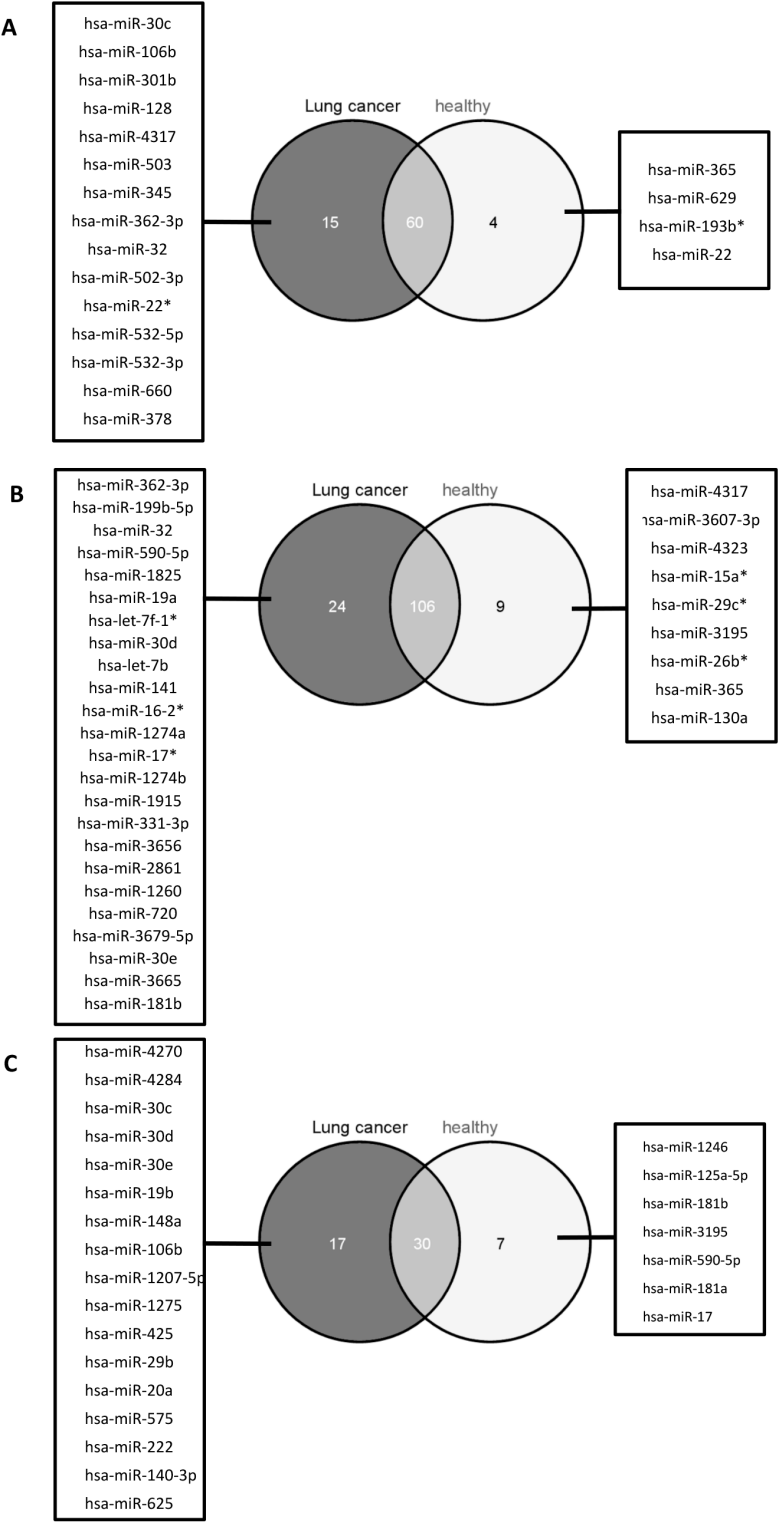
Venn diagram with the miRNAs indicative for blood cell origin, line of defense, and function We identified the miRNAs deregulated between cells with common myelod progenitor and common lyphoid progenitor **(A)**, cells of the innate immune system and the adaptive immune system **(B)**, as well as antigen presenting cells and cytotoxic cells **(C)** for lung cancer patients (dark grey circles) and healthy individuals (light gray circles), respectively. The miRNAs specific in the comparisons for lung cancer sapmles are listed in the boxes on the left, miRNAs specific in the comparisons for healthy individuals are listed in the boxes on the right. The number of miRNAs that were deregulated in lung cancer samples and healthy individuals are indicated by the overlap of the circles.

Furthermore, the comparison of antigen presenting cells (CD19+, CD14+) with cytotoxic cells (CD3+, CD56+) revealed 47 miRNAs significantly deregulated in lung cancer blood samples and 37 miRNAs in blood samples from healthy individuals. Here, the overlap was 30 miRNAs identically deregulated and 17 and 7 miRNAs were specific for lung cancer and control, respectively. The results of this analysis are visualized as venn diagram in Figure 3.

### Pathway analysis of validated target genes

In brief, we performed an over-representation analysis using GeneTrail with the validated target genes of the leukocyte subtype specific miRNAs for lung cancer and control samples, separately, and for the miRNAs that were deregulated in the different leukocyte subsets between lung cancer samples and normal control samples. KEGG pathway analysis for the miRNAs specifc for leukocyte subsets of lung cancer paptients revealed the majority of significant pathways in CD3+ T-cells. For normal controls, the majority of significant pathways were found for CD19+ B-cells. For the miRNAs deregulated between leukocyte subsets isolated from blood of lung cancer patients or healthy controls we found the most significant pathways for CD15+ granulocytes and CD19+ B-cells. Supplemental Figure 1 gives an overview of the significant KEGG pathways.

## DISCUSSION

It is already known that the cells of the immune system interact with non-immune cells to maintain tissue homeostasis [22]. A major task of the immune system is to continually recognize and remove malignant cells that arise during one's lifetime – a process called “immune surveillance” [23]. Former studies have shown that the immune status of healthy individuals differs from that of patients bearing malignant lesions. For example, T-lymphocytes in tumor patients are functionally impaired and fail to control the disease [24]. Also chronically activated innate immune cells can promote cancer development. The present study aimed to investigate the miRNome of different leukocyte subpopulations, including eosinophilic and neutrophilic granulocytes (CD15+), monocytes (CD14+), B-cells (CD19+), T-cells (CD3+), and natural killer (NK) cells (CD56+), to investigate the origin of the lung cancer specific miRNA expression pattern that is known for whole blood. To the best of our knowledge, comparable investigations have only been done on blood of healthy individuals or patients suffering from hematological diseases, so far. But there is no study investigating the effect of non-hematological diseases like lung cancer on single immune cell subsets. The present study set out to provide an overview of how much the immune cell specific miRNome is altered by the presence of a non-hematological malignancy and which cells contribute to the lung cancer specific miRNA expression pattern.

Our data clearly indicate that the different immune cell subsets have a highly specific miRNA expression pattern that is important for the regulation of the function of each immune cell. In detail, we identified immune cell type specific miRNAs in the blood of healthy individuals and lung cancer patients. Some of those miRNAs were already found by others, too [16–18]. As already shown by Allantaz et al. for blood samples of healthy individuals, the overall miRNA expression pattern was indicative for the lineage of the cell types, i.e., lymphoid or myeloid cells [16], and also indicative for the line of defense (innate or adaptive immune system), and their function (antigen presenting cells or cytotoxic cells). For the above mentioned comparisons we computed the deregulated miRNAs for healthy individuals and lung cancer patients, respectively. Here we found a high overlap of the deregulated miRNAs for healthy individuals and lung cancer samples suggesting that the specific expression pattern is not mainly influenced by lung cancer.

In spite of the above mentioned similarities between healthy individuals and lung cancer patients, we also uncovered miRNAs for each analyzed blood cell subset that were deregulated between healthy individuals and lung cancer patients. Those miRNAs were sufficient to differentiate lung cancer patients from controls with accuracies between 69% and 100%. Against our expectations, classification results for cells of the adaptive immune system, i.e., T-cells and B-cells, were worst with accuracies of only 69.23% and 76.92%, respectively. It is well known that T-cells can have opposite effects on cancer. On the one hand, anticancer T-cells can attack malignant cells and, thus, play central roles in immunity against malignant diseases. On the other hand immune suppressive cells, such as regulatory T-cells (Treg) can inhibit anticancer T-cells and, thus, are involved in tumor escape from the host immune system [24, 25]. As Treg accumulate in the peripheral circulation of cancer patients, one would normally expect a larger effect on the miRNA expression pattern of the CD3+ T-cells in blood of lung cancer patients [26]. But regarding the immune cell type specific miRNomes, we found only few lymphocyte specific miRNAs with the least differences between healthy individuals and lung cancer patients. However, KEGG-pathway over-representation analyses using validated target genes of the cell type specific miRNAs revealed the most significant KEGG pathways for CD3+ T-cells in lung cancer patient samples and for CD19+ B-cells in samples from healthy individuals. Perfect or nearly perfect classification results were obtained for cells of the innate immune system, i.e., CD14+ monocytes, CD15+ granulocytes and CD56+ NK-cells. In addition, the most cell type and lung cancer specific miRNAs were found for monocytes and granulocytes. Regarding the above mentioned deregulated miRNAs, we found only few overlaps between the cell type specific comparisons. Only one miRNA, hsa-miR-21, was upregulated in all comparisons. Interestingly, hsa-miR-21 is a well-known oncomiR and its overexpression in tissue or serum/plasma is correlated with a poor prognosis for patients with primary squamous cell lung carcinoma [27–31]. Furthermore it was recently found to be associated with increase DNA damage tolerance in lung cancer [32]. Some other miRNAs found in our study to be deregulated in certain cell types between lung cancer samples and controls were already found to be associated with lung cancer. For example, has-miR-155, that was down-regulated in monocytes of lung cancer patients in our study, was shown to be significantly reduced in the circulation of NSCLC patients compared to controls[33]. Hsa-miR-150 was down-regulated in whole blood and B-cells in the present study. This miRNA is mainly expressed in lymphocytes and an important regulator of hematopoiesis. Down-regulation of hsa-miR-150 contributes to tumorigenesis [34, 35]. However, in lung cancer tissue, controversial results were found. One study described that up-regulation of hsa-miR-150 results in lung cancer proliferation by targeting p53, but in another study it was shown that hsa-miR-150 is down-regulated in NSCLC tissue [36, 37].

Besides the miRNAs that were deregulated between lung cancer patients and controls, we also identified miRNAs that seem to be specific for one single immune cell subtype for lung cancer patients and healthy individuals, separately. The majority of specific miRNAs were found for cells with a common myeloid progenitor (monocytes and granulocytes) whereas we found only few specific miRNAs for the different types of lymphocytes (B-, T-, and NK-cells). This phenomenon was observed for both blood derived from healthy individuals as well as from lung cancer patients. In contrast, Watkins et al. found the least cell type specific miRNAs in granulocytes and monocytes and the most cell type specific miRNAs in lymphocytes. This suggests reciprocation between the amount of miRNAs found in our study and the amount of mRNAs in the study of Watkins et al. [38]. Indeed, we found cell type specific mRNAs in the Watkins study that might be potential targets of cell type specific miRNAs identified in our study.

Comparing the miRNA expression pattern of the single immune cell subsets with the whole blood miRNome suggests that the whole blood miRNome is not mirrored by a combinatorial consideration of the five investigated leukocyte subtypes. Surprisingly, we found for both control and lung cancer samples a panel of miRNAs that were not detected in any analyzed immune cell subset sample but in all whole blood samples. We also found a panel of miRNAs that were detected in more than 90% of the immune cell subset samples but not in any whole blood sample. These results suggest that there are a couple of further blood compartments other than leukocytes (e.g., erythrocytes, platelets, exosomes, etc.) that contribute to the whole blood miRNome. On the other hand, some leukocyte specific miRNAs might not reach the detection limit of the microarray in the whole blood samples, i.e., these miRNAs might be too much diluted in the whole blood samples.

## CONCLUSION

In conclusion, here we identified miRNAs specific for certain immune cell types, including eosinophilic and neutrophilic granulocytes, NK cells, monocytes, B-cells, and T-cells in lung cancer patients and healthy individuals. We further found evidence that the miRNA expression pattern depends on the cell origin (myeloid or lymphoid progenitors), the line of defense (innate or adaptive immune system), and the function (antigen presenting cells or cytotoxic cells). The overlaps in the cell type specific miRNAs between lung cancer samples and controls suggest that there is a basic miRNA expression pattern that is responsible for the specific functions of each immune cell type. However, some miRNAs differ between controls and lung cancer patients and were sufficient for highly accurate classification. In addition, from our former studies on whole blood, we know that there is a specific lung cancer miRNA signature. However, this lung cancer specific miRNA signature of whole blood could not be restricted to one certain immune cell subtype analyzed here, but in fact, seems also to be influenced by other blood constituents that have to be investigated in future studies.

## MATERIALS AND METHODS

### Blood samples

We obtained blood from 7 healthy subjects and 7 lung cancer patients drawn in EDTA Monovettes (Sarstedt) and in PAXgene Blood RNA Tubes (Beckton Dickinson). Details on the blood donors are given in Table 3. The study was approved by the local ethics comittee (01/08). We obtained informed consent from each participant.

**Table 3:** Information on all blood donors

	Lung cancer patients	healthy individuals
		
number of individuals	7	7
		
**classification**		
adenocarcinoma	5	-
squamous cell lung cancer	1	-
Small cell lung cancer	1	-
		
**gender**		
female	3	5
male	4	2
		
age, mean	64	41

### Cell sorting and RNA isolation

We isolated five different immune cell subsets from whole blood samples from seven lung cancer patients and seven healthy individuals. Protocols were optimized in order to minimize the amount of blood necessary to isolate enough cells per subtype for downstream analyses. Isolation of cell subpopulation was performed using positive selection to reduce contamination with other cells and to reach high purity of the isolated cell subsets. CD3, CD19, CD15, and CD14 positive cells were separated using non-magnetic beads and a sieve (pluriSelect), whereas CD56 positive cells were isolated using magnetic beads (Miltenyi Biotech) as non-magnetic beads were not available for this cell type. Though we used kits suitable for whole blood, we performed the separation only with leukocytes to minimize contamination with erythrocytes. Therefore, we incubated the blood with erythrocyte lysis buffer (10 mM TRIS-HCl, 165 mM NH_4_Cl, 1:3) for 12 min at RT and and washed the resulting leukocytes with PBS. The leukocytes were resuspended in the same amount of PBS as compared to the amount of blood used. The non-magnetic cell sorting was performed sequential i.e., the flowthrough of a previous separation was used as sample material for the subsequent separation. For the separation with the non-magnetic beads we used about 11 ml leukocyte suspension. After incubation with the non-magnetic beads on the pluriPlix^®^ sample mixer, the suspension was filtered through a sieve. Cells bound to the beads were retained and directly lysed on the sieve using QIAzol lysis reagent. Lysates were subsequently stored at −70°C until RNA isolation. The immune cell subsets were isolated with different incubation times in the following order: CD15 (10 min incubation), CD3 (15 min incubation), CD19 (20 min incubation), and CD14 (25 min incubation). In parallel, CD56 positive cells were isolated using magnetic beads and whole blood columns. Therefore, 3 ml leukocyte suspension was incubated for 15 min at 4°C with 150 μl Whole Blood CD56 MicroBeads according to manufacturer's instructions. The isolated CD56 positive cells were resuspended in QIAzol lysis reagent and stored at −70°C until RNA isolation. Total RNA including small RNAs was isolated from sorted cells using miRNeasy Micro Kit (Qiagen) according to manufacturer's instructions.

Total RNA including miRNA from whole blood (PAXgene Blood RNA Tubes) was isolated using the PAXgene Blood miRNA Kit (Qiagen) following the manufacturers recommendations.

Isolated RNA was stored at −80°C. RNA integrity was analyzed using Bioanalyzer 2100 (Agilent) and concentration and purity was measured using NanoDrop 2000 (Thermo Scientific).

### miRNA Microarray Analyses

The miRNA expression analysis was performed using Sure Print G3 Human v16 8×60K miRNA microarrays according to manufacturer's instruction and previously described [12]. Each array contained 40 replicates of each of the 1,205 miRNAs of miRBase v16 (http://www.mirbase.org/).

### Data analysis

Signal intensity values were extracted from the image file using Feature Extraction software (Agilent Technologies). To calculate the total expression value for each miRNA per sample we summed up the gTotalProbeSignals in the feature extraction file. Quantile normalization was applied to normalize expression values across the arrays using the preprocessCore package of the programming language R and we performed a log_2_ transformation of the data. For cluster analysis, we applied complete linkage hierarchical clustering using the Euclidian distance to compute the dissimilarity of miRNA (rows) and samples (columns) independently of each other using the normalized data. To compare expression values of miRNAs between the control and lung cancer groups, we applied the independent two-tailed t-test to find significantly deregulated miRNAs. The computed p-values were adjusted for multiple testing using the FDR (false discovery rate) approach by Benjamini and Hochberg [20, 21].

To identify blood cell type specific miRNAs, we performed t-tests for each possible pairwise combination of cell types. Then we extracted for each cell type those miRNAs that had an adjusted p-value < 0.05 and were significantly deregulated in each of the comparisons with the considered cell type. After that we removed those miRNAs from the cell type specific set that were also contained in the set of any other cell type to finally obtain five disjoint sets of miRNAs. We obtained the validated targets for those leukocyte subtype specific miRNAs from miRWalk [39] and performed with GeneTrail [40] an over-representation analysis of those targets in KEGG pathways [41] using the validated targets of the miRNA chip as reference. For creating the overview figures we further reduced the resulting significant pathways by showing only those that contained at least 10% of the target genes for the considered cell subtype.

Classification of samples using miRNA patterns was carried out using Support Vector Machines (SVM) as implemented in the R e1071 package using standard leave-one-out cross-validation.

## SUPPLEMENTARY FIGURES AND TABLES


