# Supplementary material for: What makes a blood cell based miRNA expression pattern disease specific? - A miRNome analysis of blood cell subsets in lung cancer patients and healthy controls
Source: Oncotarget. 2014 Sep 19;5(19):9484–97. doi: 10.18632/oncotarget.2419 (PMC4253448; doi:10.18632/oncotarget.2419)
Supplement: Supplementary file 1 [file oncotarget-05-9484-s001.pdf]

SUPPLEMENTARY FIGURES AND TABLES

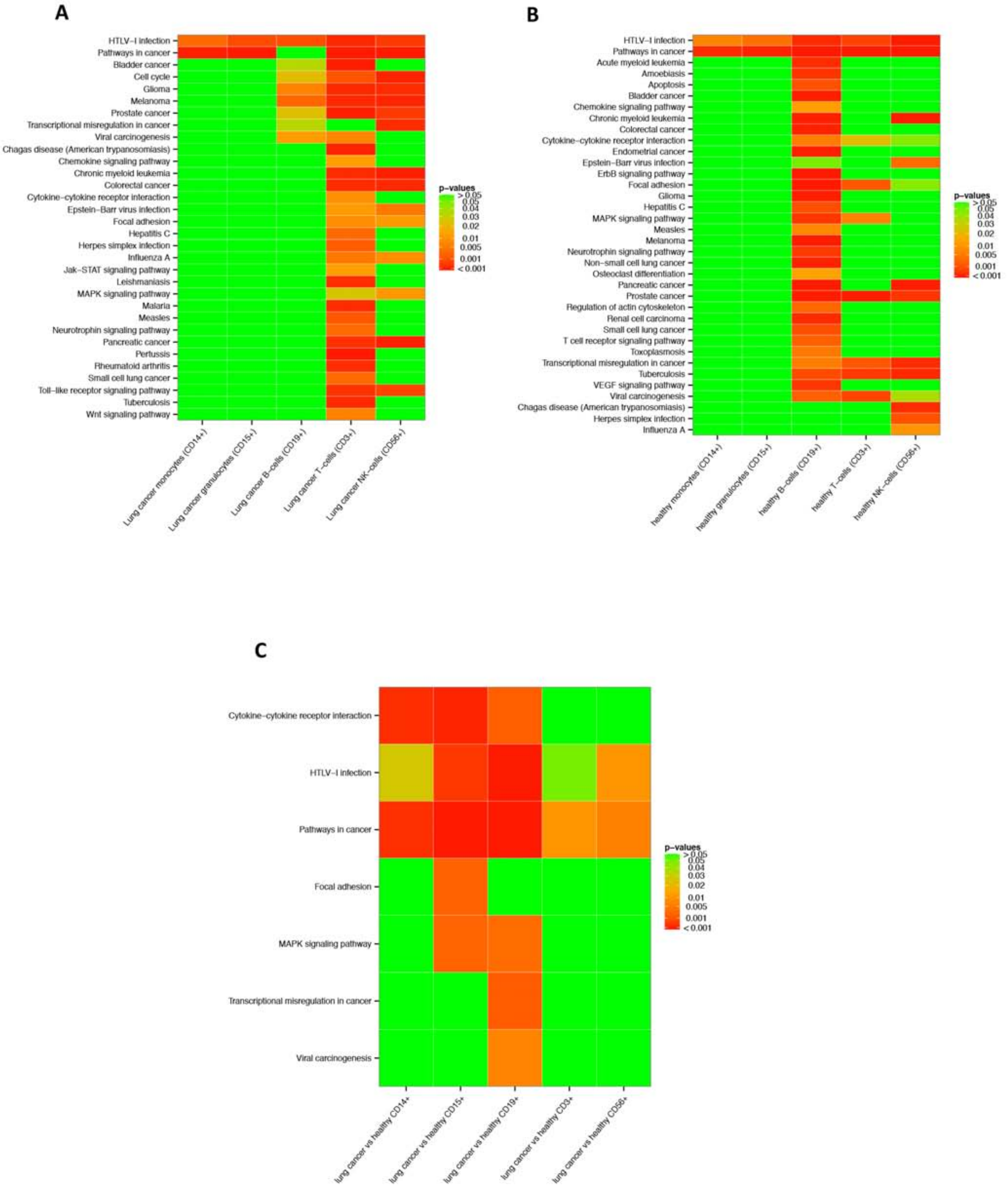

Supplemental Figure S1: Overview of KEGG pathways. Here the results of the over-representation analysis with the validated target genes of the leukocyte subtype specific miRNAs for lung cancer **(A)** and control **(B)** samples, separately, and for the miRNAs that were deregulated in the different leukocyte subsets between lung cancer samples and normal control samples **(C)**.

Supplemental Tabel S1: Cell fraction specific miRNAs exclusively differentially expressed in each cell type. This Table contains several sheets with the specific miRNAs for each cell type in controls and lung cancer patients, separately, and the intersections of deregulated miRNAs according to the adjusted t test (p-value < 0.05) between healthy individuals and lung cancer patients. 1 = specific, 0 = not specific.

	lung cancer_cd14	normal_cd14
hsa-let-7a	1	1
hsa-let-7c	1	1
hsa-let-7d	1	1
hsa-miR-1207-5p	1	0
hsa-miR-1225-5p	1	0
hsa-miR-130b	1	1
hsa-miR-142-5p	1	1
hsa-miR-17	1	1
hsa-miR-18a	1	0
hsa-miR-18b	1	0
hsa-miR-1915	1	1
hsa-miR-19b	1	0
hsa-miR-21	1	1
hsa-miR-22*	1	0
hsa-miR-221	1	1
hsa-miR-26b*	1	0
hsa-miR-2861	1	0
hsa-miR-29b	1	1
hsa-miR-29c	1	1
hsa-miR-30d	1	1
hsa-miR-324-5p	1	1
hsa-miR-326	1	1
hsa-miR-331-3p	1	1
hsa-miR-345	1	0
hsa-miR-362-3p	1	1
hsa-miR-362-5p	1	1
hsa-miR-3656	1	0
hsa-miR-3665	1	0
hsa-miR-378	1	1
hsa-miR-378*	1	0
hsa-miR-421	1	0
hsa-miR-423-3p	1	0
hsa-miR-484	1	1
hsa-miR-500a	1	0

	lung cancer_cd14	normal_cd14
hsa-miR-500a*	1	1
hsa-miR-501-3p	1	0
hsa-miR-502-3p	1	1
hsa-miR-502-5p	1	0
hsa-miR-532-3p	1	1
hsa-miR-532-5p	1	1
hsa-miR-638	1	0
hsa-miR-660	1	1
hsa-miR-664	1	0
hsa-miR-762	0	1
hsa-miR-92a	0	1
hsa-miR-98	1	0

	lung cancer_cd15	normal_cd15
hsa-let-7f-1*	1	0
hsa-let-7i	1	0
hsa-let-7i*	1	1
hsa-miR-101*	1	1
hsa-miR-1234	0	1
hsa-miR-1237	1	0
hsa-miR-1238	0	1
hsa-miR-1260	0	1
hsa-miR-1260b	1	1
hsa-miR-1274a	1	0
hsa-miR-128	0	1
hsa-miR-1280	0	1
hsa-miR-1305	1	1
hsa-miR-1306	1	1
hsa-miR-135a*	1	0
hsa-miR-143	1	1
hsa-miR-145	1	1
hsa-miR-145*	1	1
hsa-miR-148a	0	1
hsa-miR-1537	0	1
hsa-miR-16-2*	1	0
hsa-miR-181c	1	0
hsa-miR-181c*	1	1
hsa-miR-1825	0	1
hsa-miR-1914*	1	0
hsa-miR-193a-5p	1	1
hsa-miR-197	1	1
hsa-miR-1972	1	1
hsa-miR-199a-3p	0	1
hsa-miR-202	1	0
hsa-miR-20b	1	1
hsa-miR-2115	1	1
hsa-miR-219-5p	1	0
hsa-miR-22	0	1
hsa-miR-221*	1	0
hsa-miR-2355-5p	1	1
hsa-miR-29a*	1	0

	lung cancer_cd15	normal_cd15
hsa-miR-30b	0	1
hsa-miR-30e	1	1
hsa-miR-30e*	0	1
hsa-miR-3188	0	1
hsa-miR-3198	1	1
hsa-miR-32	1	0
hsa-miR-320c	1	1
hsa-miR-320d	1	1
hsa-miR-320e	1	1
hsa-miR-324-3p	1	0
hsa-miR-330-3p	1	0
hsa-miR-338-3p	0	1
hsa-miR-365	1	0
hsa-miR-3663-3p	1	1
hsa-miR-3679-3p	1	0
hsa-miR-424*	1	1
hsa-miR-4270	1	1
hsa-miR-4281	0	1
hsa-miR-4284	1	1
hsa-miR-4313	0	1
hsa-miR-4317	1	1
hsa-miR-4323	1	1
hsa-miR-450a	0	1
hsa-miR-491-5p	0	1
hsa-miR-505	0	1
hsa-miR-505*	1	1
hsa-miR-513a-5p	1	0
hsa-miR-519e*	1	0
hsa-miR-542-3p	1	1
hsa-miR-542-5p	1	1
hsa-miR-545	1	1
hsa-miR-548e	1	0
hsa-miR-550a*	1	1
hsa-miR-582-3p	1	1
hsa-miR-624*	1	0
hsa-miR-627	1	1
hsa-miR-628-3p	1	1

(*Continued*)

	lung cancer_cd15	normal_cd15
hsa-miR-628-5p	1	1
hsa-miR-629	1	1
hsa-miR-629*	1	1
hsa-miR-642b	1	0
hsa-miR-769-3p	1	0
hsa-miR-769-5p	1	1
hsa-miR-99b	0	1

	lung cancer_cd19	normal_cd19
hsa-miR-126	0	1
hsa-miR-3907	1	0
hsa-miR-423-5p	1	0
hsa-miR-4261	1	1
hsa-miR-574-5p	0	1
hsa-miR-940	1	0

	lung cancer_cd3	normal_cd3
hsa-miR-125b	0	1
hsa-miR-1275	1	0
hsa-miR-192	0	1
hsa-miR-194	1	1
hsa-miR-215	0	1
hsa-miR-31	1	1
hsa-miR-342-5p	1	0
hsa-miR-3607-3p	1	0
hsa-miR-564	1	0
hsa-miR-7-1*	0	1
hsa-miR-874	1	1
hsa-miR-99a	0	1

	lung cancer_cd56	normal_cd56
hsa-miR-10a	0	1
hsa-miR-139-3p	0	1
hsa-miR-148b	1	0
hsa-miR-152	1	1
hsa-miR-181a-2*	1	1
hsa-miR-215	1	0
hsa-miR-23b	0	1
hsa-miR-24	1	0
hsa-miR-27a	0	1
hsa-miR-27b	0	1
hsa-miR-3195	0	1
hsa-miR-340	0	1
hsa-miR-365	0	1
hsa-miR-873	0	1

	normal_cd14	normal_cd15	normal_cd19	normal_cd3	normal_cd56
hsa-let-7a	1	0	0	0	0
hsa-let-7c	1	0	0	0	0
hsa-let-7d	1	0	0	0	0
hsa-let-7i*	0	1	0	0	0
hsa-miR-101*	0	1	0	0	0
hsa-miR-10a	0	0	0	0	1
hsa-miR-1234	0	1	0	0	0
hsa-miR-1238	0	1	0	0	0
hsa-miR-125b	0	0	0	1	0
hsa-miR-126	0	0	1	0	0
hsa-miR-1260	0	1	0	0	0
hsa-miR-1260b	0	1	0	0	0
hsa-miR-128	0	1	0	0	0
hsa-miR-1280	0	1	0	0	0
hsa-miR-1305	0	1	0	0	0
hsa-miR-1306	0	1	0	0	0
hsa-miR-130b	1	0	0	0	0
hsa-miR-139-3p	0	0	0	0	1
hsa-miR-142-5p	1	0	0	0	0
hsa-miR-143	0	1	0	0	0
hsa-miR-145	0	1	0	0	0
hsa-miR-145*	0	1	0	0	0
hsa-miR-148a	0	1	0	0	0
hsa-miR-152	0	0	0	0	1
hsa-miR-1537	0	1	0	0	0
hsa-miR-17	1	0	0	0	0
hsa-miR-181a-2*	0	0	0	0	1
hsa-miR-181c*	0	1	0	0	0
hsa-miR-1825	0	1	0	0	0
hsa-miR-1915	1	0	0	0	0
hsa-miR-192	0	0	0	1	0
hsa-miR-193a-5p	0	1	0	0	0
hsa-miR-194	0	0	0	1	0
hsa-miR-197	0	1	0	0	0
hsa-miR-1972	0	1	0	0	0
hsa-miR-199a-3p	0	1	0	0	0
hsa-miR-20b	0	1	0	0	0

	normal_cd14	normal_cd15	normal_cd19	normal_cd3	normal_cd56
hsa-miR-21	1	0	0	0	0
hsa-miR-2115	0	1	0	0	0
hsa-miR-215	0	0	0	1	0
hsa-miR-22	0	1	0	0	0
hsa-miR-221	1	0	0	0	0
hsa-miR-2355-5p	0	1	0	0	0
hsa-miR-23b	0	0	0	0	1
hsa-miR-27a	0	0	0	0	1
hsa-miR-27b	0	0	0	0	1
hsa-miR-29b	1	0	0	0	0
hsa-miR-29c	1	0	0	0	0
hsa-miR-30b	0	1	0	0	0
hsa-miR-30d	1	0	0	0	0
hsa-miR-30e	0	1	0	0	0
hsa-miR-30e*	0	1	0	0	0
hsa-miR-31	0	0	0	1	0
hsa-miR-3188	0	1	0	0	0
hsa-miR-3195	0	0	0	0	1
hsa-miR-3198	0	1	0	0	0
hsa-miR-320c	0	1	0	0	0
hsa-miR-320d	0	1	0	0	0
hsa-miR-320e	0	1	0	0	0
hsa-miR-324-5p	1	0	0	0	0
hsa-miR-326	1	0	0	0	0
hsa-miR-331-3p	1	0	0	0	0
hsa-miR-338-3p	0	1	0	0	0
hsa-miR-340	0	0	0	0	1
hsa-miR-362-3p	1	0	0	0	0
hsa-miR-362-5p	1	0	0	0	0
hsa-miR-365	0	0	0	0	1
hsa-miR-3663-3p	0	1	0	0	0
hsa-miR-378	1	0	0	0	0
hsa-miR-424*	0	1	0	0	0
hsa-miR-4261	0	0	1	0	0
hsa-miR-4270	0	1	0	0	0
hsa-miR-4281	0	1	0	0	0
hsa-miR-4284	0	1	0	0	0

(*Continued*)

	normal_cd14	normal_cd15	normal_cd19	normal_cd3	normal_cd56
hsa-miR-4313	0	1	0	0	0
hsa-miR-4317	0	1	0	0	0
hsa-miR-4323	0	1	0	0	0
hsa-miR-450a	0	1	0	0	0
hsa-miR-484	1	0	0	0	0
hsa-miR-491-5p	0	1	0	0	0
hsa-miR-500a*	1	0	0	0	0
hsa-miR-502-3p	1	0	0	0	0
hsa-miR-505	0	1	0	0	0
hsa-miR-505*	0	1	0	0	0
hsa-miR-532-3p	1	0	0	0	0
hsa-miR-532-5p	1	0	0	0	0
hsa-miR-542-3p	0	1	0	0	0
hsa-miR-542-5p	0	1	0	0	0
hsa-miR-545	0	1	0	0	0
hsa-miR-550a*	0	1	0	0	0
hsa-miR-574-5p	0	0	1	0	0
hsa-miR-582-3p	0	1	0	0	0
hsa-miR-627	0	1	0	0	0
hsa-miR-628-3p	0	1	0	0	0
hsa-miR-628-5p	0	1	0	0	0
hsa-miR-629	0	1	0	0	0
hsa-miR-629*	0	1	0	0	0
hsa-miR-660	1	0	0	0	0
hsa-miR-7-1*	0	0	0	1	0
hsa-miR-762	1	0	0	0	0
hsa-miR-769-5p	0	1	0	0	0
hsa-miR-873	0	0	0	0	1
hsa-miR-874	0	0	0	1	0
hsa-miR-92a	1	0	0	0	0
hsa-miR-99a	0	0	0	1	0
hsa-miR-99b	0	1	0	0	0

	lung cancer_cd14	lung cancer_cd15	lung cancer_cd19	lung cancer_cd3	lung cancer_cd56
hsa-let-7a	1	0	0	0	0
hsa-let-7c	1	0	0	0	0
hsa-let-7d	1	0	0	0	0
hsa-let-7f-1*	0	1	0	0	0
hsa-let-7i	0	1	0	0	0
hsa-let-7i*	0	1	0	0	0
hsa-miR-101*	0	1	0	0	0
hsa-miR-1207-5p	1	0	0	0	0
hsa-miR-1225-5p	1	0	0	0	0
hsa-miR-1237	0	1	0	0	0
hsa-miR-1260b	0	1	0	0	0
hsa-miR-1274a	0	1	0	0	0
hsa-miR-1275	0	0	0	1	0
hsa-miR-1305	0	1	0	0	0
hsa-miR-1306	0	1	0	0	0
hsa-miR-130b	1	0	0	0	0
hsa-miR-135a*	0	1	0	0	0
hsa-miR-142-5p	1	0	0	0	0
hsa-miR-143	0	1	0	0	0
hsa-miR-145	0	1	0	0	0
hsa-miR-145*	0	1	0	0	0
hsa-miR-148b	0	0	0	0	1
hsa-miR-152	0	0	0	0	1
hsa-miR-16-2*	0	1	0	0	0
hsa-miR-17	1	0	0	0	0
hsa-miR-181a-2*	0	0	0	0	1
hsa-miR-181c	0	1	0	0	0
hsa-miR-181c*	0	1	0	0	0
hsa-miR-18a	1	0	0	0	0
hsa-miR-18b	1	0	0	0	0
hsa-miR-1914*	0	1	0	0	0
hsa-miR-1915	1	0	0	0	0
hsa-miR-193a-5p	0	1	0	0	0
hsa-miR-194	0	0	0	1	0
hsa-miR-197	0	1	0	0	0
hsa-miR-1972	0	1	0	0	0
hsa-miR-19b	1	0	0	0	0

(*Continued*)

	lung cancer_cd14	lung cancer_cd15	lung cancer_cd19	lung cancer_cd3	lung cancer_cd56
hsa-miR-202	0	1	0	0	0
hsa-miR-20b	0	1	0	0	0
hsa-miR-21	1	0	0	0	0
hsa-miR-2115	0	1	0	0	0
hsa-miR-215	0	0	0	0	1
hsa-miR-219-5p	0	1	0	0	0
hsa-miR-22*	1	0	0	0	0
hsa-miR-221	1	0	0	0	0
hsa-miR-221*	0	1	0	0	0
hsa-miR-2355-5p	0	1	0	0	0
hsa-miR-24	0	0	0	0	1
hsa-miR-26b*	1	0	0	0	0
hsa-miR-2861	1	0	0	0	0
hsa-miR-29a*	0	1	0	0	0
hsa-miR-29b	1	0	0	0	0
hsa-miR-29c	1	0	0	0	0
hsa-miR-30d	1	0	0	0	0
hsa-miR-30e	0	1	0	0	0
hsa-miR-31	0	0	0	1	0
hsa-miR-3198	0	1	0	0	0
hsa-miR-32	0	1	0	0	0
hsa-miR-320c	0	1	0	0	0
hsa-miR-320d	0	1	0	0	0
hsa-miR-320e	0	1	0	0	0
hsa-miR-324-3p	0	1	0	0	0
hsa-miR-324-5p	1	0	0	0	0
hsa-miR-326	1	0	0	0	0
hsa-miR-330-3p	0	1	0	0	0
hsa-miR-331-3p	1	0	0	0	0
hsa-miR-342-5p	0	0	0	1	0
hsa-miR-345	1	0	0	0	0
hsa-miR-3607-3p	0	0	0	1	0
hsa-miR-362-3p	1	0	0	0	0
hsa-miR-362-5p	1	0	0	0	0
hsa-miR-365	0	1	0	0	0
hsa-miR-3656	1	0	0	0	0
hsa-miR-3663-3p	0	1	0	0	0

	lung cancer_cd14	lung cancer_cd15	lung cancer_cd19	lung cancer_cd3	lung cancer_cd56
hsa-miR-3665	1	0	0	0	0
hsa-miR-3679-3p	0	1	0	0	0
hsa-miR-378	1	0	0	0	0
hsa-miR-378*	1	0	0	0	0
hsa-miR-3907	0	0	1	0	0
hsa-miR-421	1	0	0	0	0
hsa-miR-423-3p	1	0	0	0	0
hsa-miR-423-5p	0	0	1	0	0
hsa-miR-424*	0	1	0	0	0
hsa-miR-4261	0	0	1	0	0
hsa-miR-4270	0	1	0	0	0
hsa-miR-4284	0	1	0	0	0
hsa-miR-4317	0	1	0	0	0
hsa-miR-4323	0	1	0	0	0
hsa-miR-484	1	0	0	0	0
hsa-miR-500a	1	0	0	0	0
hsa-miR-500a*	1	0	0	0	0
hsa-miR-501-3p	1	0	0	0	0
hsa-miR-502-3p	1	0	0	0	0
hsa-miR-502-5p	1	0	0	0	0
hsa-miR-505*	0	1	0	0	0
hsa-miR-513a-5p	0	1	0	0	0
hsa-miR-519e*	0	1	0	0	0
hsa-miR-532-3p	1	0	0	0	0
hsa-miR-532-5p	1	0	0	0	0
hsa-miR-542-3p	0	1	0	0	0
hsa-miR-542-5p	0	1	0	0	0
hsa-miR-545	0	1	0	0	0
hsa-miR-548e	0	1	0	0	0
hsa-miR-550a*	0	1	0	0	0
hsa-miR-564	0	0	0	1	0
hsa-miR-582-3p	0	1	0	0	0
hsa-miR-624*	0	1	0	0	0
hsa-miR-627	0	1	0	0	0
hsa-miR-628-3p	0	1	0	0	0
hsa-miR-628-5p	0	1	0	0	0
hsa-miR-629	0	1	0	0	0

(*Continued*)

	lung cancer_cd14	lung cancer_cd15	lung cancer_cd19	lung cancer_cd3	lung cancer_cd56
hsa-miR-629*	0	1	0	0	0
hsa-miR-638	1	0	0	0	0
hsa-miR-642b	0	1	0	0	0
hsa-miR-660	1	0	0	0	0
hsa-miR-664	1	0	0	0	0
hsa-miR-769-3p	0	1	0	0	0
hsa-miR-769-5p	0	1	0	0	0
hsa-miR-874	0	0	0	1	0
hsa-miR-940	0	0	1	0	0
hsa-miR-98	1	0	0	0	0
